# Redox Stability Optimization in Anode-Supported Solid Oxide Fuel Cells

**DOI:** 10.3390/ma17133257

**Published:** 2024-07-02

**Authors:** Yu Wang, Ming Song

**Affiliations:** 1 College of Transportation, Shandong University of Science and Technology, Qingdao 266555, China; skdwangyu@sdust.edu.cn; 2 Department of Engineering Mechanics, College of Pipeline and Civil Engineering, China University of Petroleum (East China), Qingdao 266555, China

**Keywords:** solid oxide fuel cell, redox, stress, anode functional layer, thickness

## Abstract

For Ni-YSZ anode-supported solid oxide fuel cells (SOFCs), the main drawback is that they are susceptible to reducing and oxidizing atmosphere changes because of the Ni/NiO volume variation. The anode expansion upon oxidation can cause significant stresses in the cell, eventually leading to failure. In order to improve the redox stability, an analytical model is developed to study the effect of anode structure on redox stability. Compared with the SOFC without AFL, the tensile stresses in the electrolyte and cathode of SOFC with an anode functional layer (AFL) after anode oxidation are increased by 27.07% and 20.77%, respectively. The thickness of the anode structure has a great influence on the structure’s stability. Therefore, the influence of anode thickness and AFL thickness on the stress in these two structures after oxidation is also discussed. The thickness of the anode substrate plays a more important role in the SOFC without AFL than in the SOFC with AFL. By increasing the thickness of the anode substrate, the stresses in the electrolyte and cathode decrease. This method provides a theoretical basis for the design of a reliable SOFC in the redox condition and will be more reliable with more experimental proofs in the future.

## 1. Introduction

Over the last decades, solid oxide fuel cells (SOFCs) have received sustained attention for the use of a promising medium for the conversion of chemical energy into electrical power [1]. SOFCs have the powerful ability to work at various anode gas compositions, including methane, methanol, carbon monoxide, biomass sources, hydrogen, etc. [2,3,4]. Despite the advantages of high efficiency, environmental friendliness, and fuel flexibility, structure stability should be ensured for the practical application of SOFCs, which usually comprise anode, electrolyte, and cathode layers [5].

The anode-supported SOFC is the most prevalent at present due to low ohmic losses in the electrolyte and high power densities. But the lack of redox stability has been an obstacle to its commercialization [6]. The generally used anode material is nickel-doped yttria-stabilized zirconia (Ni-YSZ). The Ni-based anode material is in the oxidized state (NiO-YSZ) after fabrication and turns to the reduced state after contacting with the fuel (H_2_) in operation at high temperatures. Either fuel supply interruption, seal leakage, or system shutdown could lead to the re-oxidation of the anode [7,8]. The re-oxidation of the anode degrades not only the electrical performance but also the mechanical strength. Timurkutluk et al. [9] found that after a single redox cycle, the mechanical properties of the cell decreased by around 50%, while the same cell showed only around 10% electrochemical performance loss. We [10] found the thermal stresses and failure probabilities of the cell are increased by re-oxidation because of the large volume expansion of the anode. If the anode expansion strain is more than 0.09% and 0.15%, cracks will occur in the cathode and the electrolyte [11]. Therefore, when determining the structure of the cell, not only the electrochemical performance but also the redox stability must be considered.

In order to improve the electrochemical performance, an anode functional layer (AFL) is usually introduced between the anode support and the electrolyte [12]. Several techniques for the preparation of AFLs have been developed for commercial application [13,14]. Therefore, the anode is composed of two parts: the electrochemical active part, i.e., the AFL, and the porous anode substrate. Because the thickness of the AFL is thin (~10 μm) compared to the anode substrate thickness (~400 μm), the effect of the AFL is usually ignored during the calculation of the stresses in the cell [15]. However, the AFL has a dense and finer microstructure than the anode substrate in order to increase the triple phase boundaries, the Young’s modulus of the AFL is rather large. Most importantly, porosity is identified as an important microstructural parameter related to dimensional and structural stability during redox cycles. Pihlatie et al. found that a decrease in porosity increased the expansion during redox cycling [16]. As the AFL has a lower porosity than the anode substrate, a higher expansion strain is yielded during re-oxidation, leading to a higher failure risk. Waldbillig et al. [17] found that coarse-structured anode samples similar to a typical anode substrate experience no volume change upon reduction or re-oxidation; however, fine-grained samples similar to an AFL increase in volume by 0.9–2.5% after re-oxidation. The transformation of Ni to NiO in the course of redox cycling is followed by a gradual change in the morphology of Ni particles, which in turn causes a lowering of the Young’s modulus and strength of the SOFC anode layer [18,19]. Kim et al. [20] focused on the three-dimensional thermal stress analysis of the re-oxidized Ni-YSZ AFL in SOFCs and found the stress near the electrolyte–anode boundary is more severe compared with the anode and the electrolyte regions, which causes delamination between the electrolyte and the anode support, leading to eventual mechanical fracture. Malzbender et al. [21] found that re-oxidation causes a larger final curvature by taking the AFL into consideration. But the effect of the AFL on stresses was not investigated.

In conclusion, the redox effect on stress has been investigated by many researchers. However, the effect of AFL has not been considered yet. In the present work, an analytical model is developed to investigate the effect of anode structure on the redox stability of SOFCs. The effect of the AFL on stress is studied by comparing the stress distribution of SOFCs with an AFL and without an AFL. Moreover, the effects of the thickness of the AFL and anode substrate on the stress after re-oxidation are discussed.

## 2. Analytical Model

**Figure 1:**
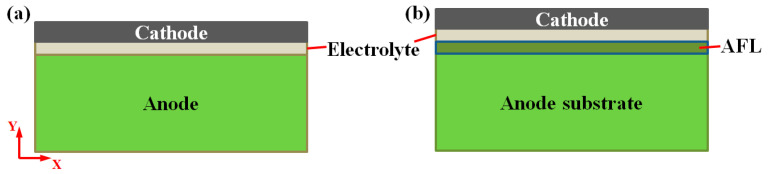
Schematic of layered SOFC without (**a**) and with AFL (**b**).

The typical anode-supported SOFC used widely in simulation is divided into three layers: the anode, electrolyte, and cathode layers (see Figure 1a). The thickness and material of each component are listed in Table 1 [10]. It should be noted that, generally, there is a diffusion barrier layer with the material of GDC between the electrolyte and cathode. However, due to the relatively thin thickness of the diffusion barrier layer (around 2 µm) compared to other layers, it can generally be omitted in the simulation calculations and considered part of the cathode [22].

In this study, a model of SOFC with an AFL on the anode substrate is developed to study the effect of the AFL on redox stability. As shown in Figure 1b, the anode substrate, AFL, electrolyte, and cathode are bonded together and have a thickness of *t_s_*, *t_f_*, *t_e,_* and *t_c_*, respectively. The *x*-axis is at the interface between the AFL layer and the anode substrate. The symbols *E_i_*, *υ_i,_* and *α_i_* represent Young’s modulus, Poisson’s ratio, and thermal expansion coefficient (CTE), respectively. They are assumed to be isotropic in each layer. The temperature-dependent mechanical properties are listed in Table 2 [23,24,25,26].

The materials were assumed to be stress-free at an initial temperature (T = 1200 °C) [27]; then, the cell was cooled down to room temperature (T = 20 °C). And the as-fabricated stresses were induced during this process. Then, the SOFC was heated to 800 °C for operation, and hydrogen was introduced into the anode. During this period, the NiO-YSZ anode substrate and AFL were reduced to Ni-YSZ. During the operation, the reduced anode substrate and AFL were re-oxidized to NiO-YSZ because of the accidental appearance of air or oxygen. The temperature was assumed to be uniform throughout the layers, so the effects of temperature gradient were not taken into account. The edge effects and singularities are neglected in this study. Based on the classical plate theory analysis, the strain *ε*(*y*,*t*) is expressed as follows:

(1) $$\varepsilon \left(y,t\right)=\varepsilon_{0}\left(t\right)+k\left(t\right)y$$

where *ε*_0_(*t*) is the strain at the interface between the anode substrate and the AFL (*y* = 0) and *k*(*t*) is the curvature. The equal biaxial stresses in different layers of the disk cell are as follows:

(2) $$\varepsilon_{i}^{th}=\alpha_{i}\Delta T$$

(3) $$\sigma_{i}\left(y,t\right)=\frac{E_{i}}{1-\nu_{i}}\left[\varepsilon_{i}\left(y,t\right)-\varepsilon_{i}^{th}-\varepsilon_{i}^{ox}\right]$$

where *ε_i_^ox^* is the oxidation strain. The oxidation strains of the anode substrate and the AFL are assumed to be 0.35% and 1.19%, as used in Ref. [28].

In order to satisfy the equilibrium condition, the summation of in-plane forces and moments in the whole system should be zero, as follows [29]:

(4) $$\int_{-t_{s}}^{0}\sigma_{s}\left(y,t\right)dy+\int_{0}^{t_{f}}\sigma_{f}\left(y,t\right)dy+\int_{t_{f}}^{t_{f}+t_{e}}\sigma_{e}\left(y,t\right)dy+\int_{t_{f}+t_{e}}^{t_{f}+t_{e}+t_{c}}\sigma_{c}\left(y,t\right)dy=0$$

(5) $$\int_{-t_{s}}^{0}\sigma_{s}\left(y,t\right)ydy+\int_{0}^{t_{f}}\sigma_{f}\left(y,t\right)ydy+\int_{t_{f}}^{t_{f}+t_{e}}\sigma_{e}\left(y,t\right)ydy+\int_{t_{f}+t_{e}}^{t_{f}+t_{e}+t_{c}}\sigma_{c}\left(y,t\right)ydy=0$$

## 3. Results and Discussion

### 3.1. Stress Evolution during Redox

**Figure 2:**
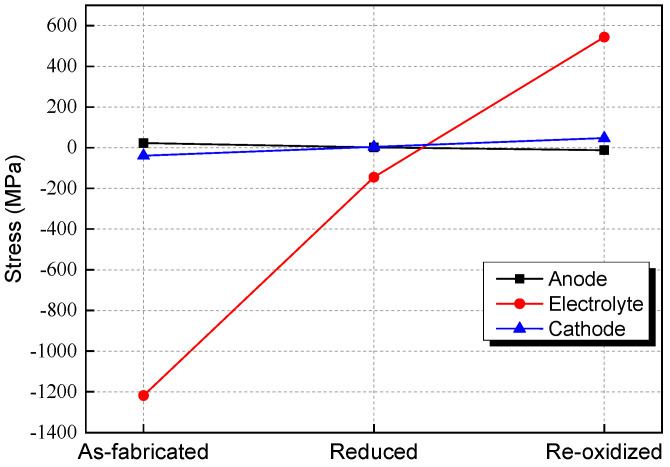
Stress after fabrication, reduction, and re-oxidation in SOFC without AFL.

Figure 2 shows the stresses of the electrolyte, cathode, and anode after the fabrication, reduction, and re-oxidation of the SOFC without an AFL. After fabrication, different residual stresses are caused in each layer due to CTE mismatch. The compressive residual stresses in the electrolyte and cathode are 1218.5 and 39.36 MPa, respectively. The residual stress in the anode is tensile and is 22.93 MPa. After the reduction of the anode at 800 °C, the stresses in the SOFC are decreased greatly. The compressive stresses in the electrolyte and cathode are decreased to 144.92 MPa. And the tensile stress in the anode is decreased to 2.12 MPa. The compressive stress in the cathode layer is turned into tensile stress and is 4.38 MPa. After the re-oxidation, the tensile stress in the cathode is increased to 47.19 MPa. The compressive stress in the electrolyte layer is turned into tensile stress and is 543.59 MPa, and the stress in the anode changes from tensile to compressive and is 12.21 MPa. This is because the volume expansion of the anode substrate in the re-oxidation process forces the electrolyte layer and the cathode layer to produce tensile deformation, which causes great tensile stress. Due to the deformation compatibility of the multilayer structure, compressive stress is generated in the anode.

After fabrication, the stresses in the anode and cathode are lower than their fracture strengths. But large compressive stress is caused in the electrolyte layer, which is beyond the compressive strength (around 1 GPa) and would lead to lamination of the cell. When the temperature is heated to 800 °C, the stresses in the anode, electrolyte, and cathode experience a reduction of 90.71%, 88.11%, and 88.87%, respectively. This is not only due to the reduction in the thermal expansion deformation of each layer but also due to the reduction in the anode elastic modulus caused by the reduction in porosity during the reduction process [10]. After the re-oxidation of the anode, the stresses of the electrolyte layer and the cathode layer both exceed their fracture strengths, which are about 154 and 75 MPa (see Table 3), respectively, leading to the fracture of the cell [30]. It should be noted that the thicknesses of the electrode layers in Table 3 are close to those used in calculations. Otherwise, the conditions would not be good for comparison.

**Figure 3:**
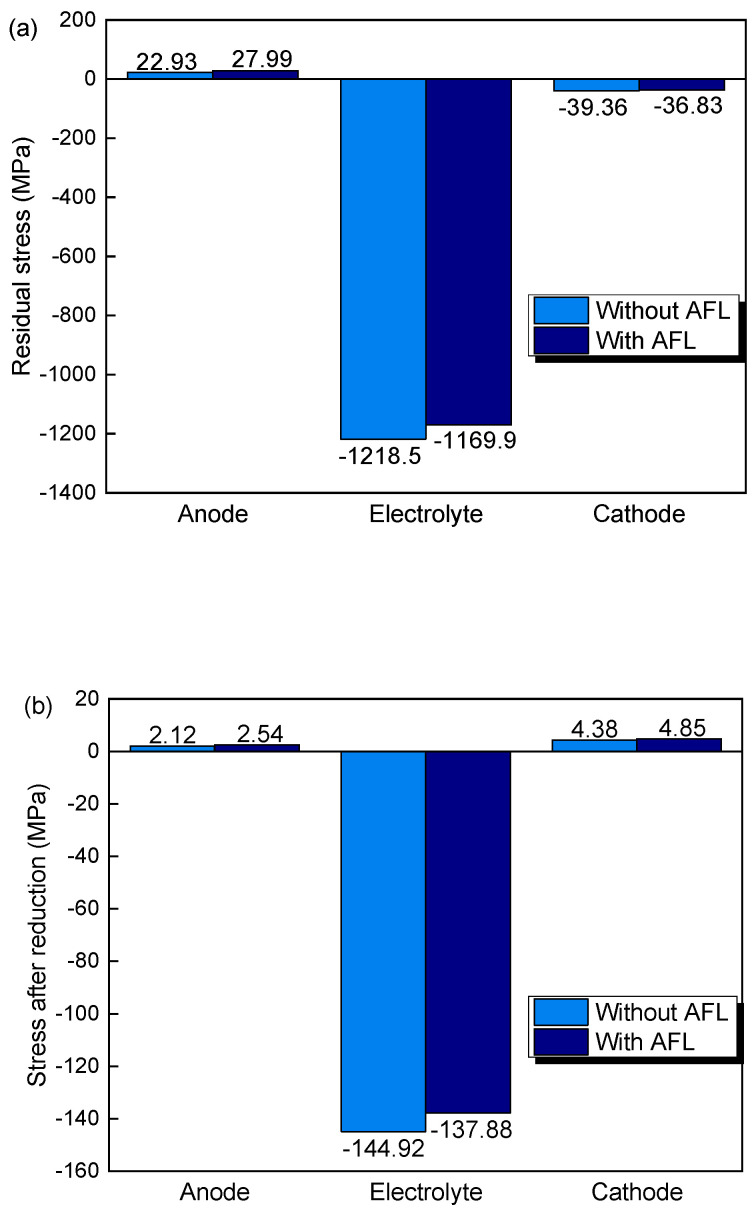

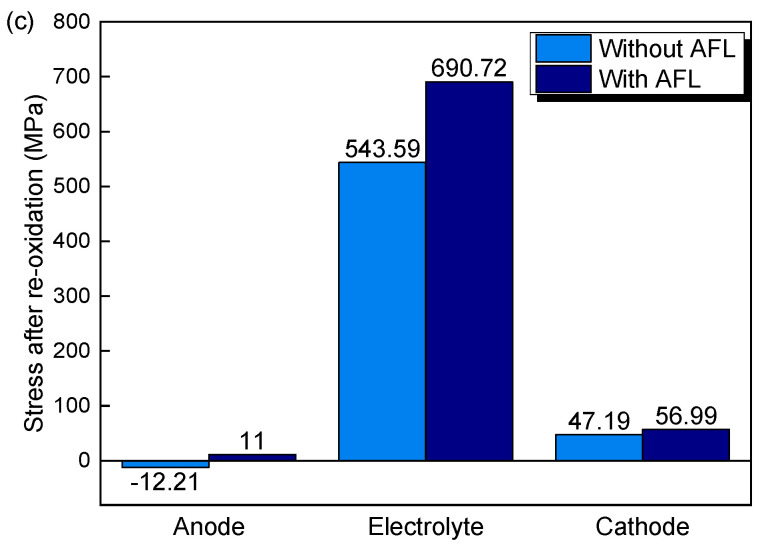
Stresses (**a**) at as-fabricated state, (**b**) after reduction, and (**c**) after re-oxidation in SOFC with and without AFL.

Figure 3 shows the stresses of the anode, electrolyte, cathode, and AFL at fabrication and after the anode reduction and re-oxidation in SOFCs with and without an AFL. After fabrication, as shown in Figure 3a, the compressive stresses of the electrolyte and cathode with an AFL are 1169.9 and 36.83 MPa and are decreased by 3.99% and 6.43%, respectively, compared with those without an AFL, while the tensile stress in the anode with an AFL is 27.99 MPa, which is 22.07% larger than that of the anode without an AFL. After the reduction, the compressive stress of electrolytes with an AFL is 137.88 MPa, which is decreased by 4.86% compared with those without an AFL, while the tensile stresses in the cathode and anode are increased by 10.73% and 19.81%, respectively, compared with those without an AFL. After the re-oxidation of the anode, the tensile stresses of the electrolyte layer and the cathode layer with an AFL are 690.72 and 56.99 MPa, which are increased by 27.07% and 20.77%, respectively, compared with those without an AFL. The stress is tensile in the anode with an AFL but compressive in the anode without an AFL. It can be seen that the stress in the AFL remains compressive but might exceed its compressive fracture strength after the re-oxidation.

It can be seen that the stress in the electrolyte of the SOFC with an AFL is smaller than that in those without an AFL in the as-fabricated SOFC and after reduction, but larger than that in those without an AFL after re-oxidation. This is because of the larger oxidation strain of the AFL than that of the anode substrate, thereby imposing larger tensile stresses on the electrolyte layer and the cathode layer. As the stress in the anode is rather small, only the stresses in the electrolyte and cathode are investigated in the following investigation.

**Figure 4:**
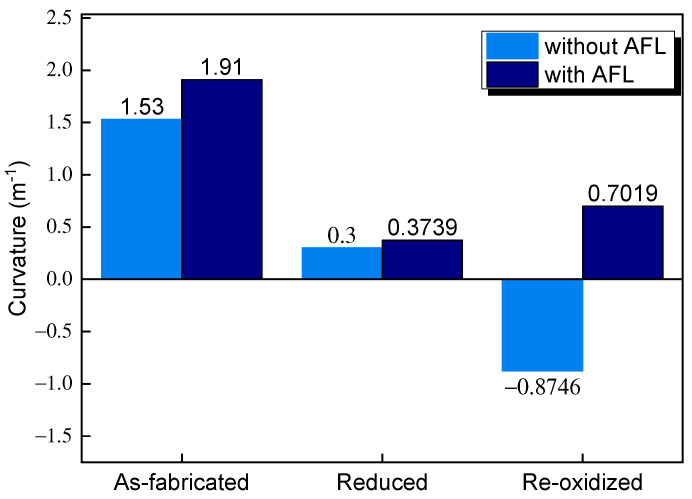
Curvatures of SOFC with and without AFL at as-fabricated state and after reduction and re-oxidation.

Figure 4 shows the curvatures of the SOFC with and without an AFL at an as-fabricated state, after the reduction and re-oxidation of the anode. The curvatures are largest after fabrication and decrease after reduction. The difference between the curvatures of the SOFC with and without an AFL is minimum after the reduction and maximum after re-oxidation. Especially, the bending directions of the SOFC with and without an AFL are opposite, indicating the great influence of the AFL on re-oxidation.

### 3.2. The Effects of AFL and Anode Substrate Thickness on Stress after Re-Oxidation

The contribution of the anode substrate to the strength of the anode-supported SOFC is significant because of its rather thick thickness as a support. The re-oxidation of the anode causes large tensile stress in the cathode and electrolyte, leading to the fracture and even failure of the cell. The AFL has a great influence on redox stability. By adding an AFL on the anode substrate, the tensile stresses of the electrolyte and cathode are increased by 27.14% and 25.06%, respectively, compared with those without an AFL, increasing the failure risk. The crack of the electrolyte may result in fuel leakage across the cathode and an associated reaction with no generation of electricity [32]. Therefore, the effects of the AFL and anode substrate thickness on stress after re-oxidation are discussed to decrease the stresses in the electrolyte and cathode.

**Figure 5:**
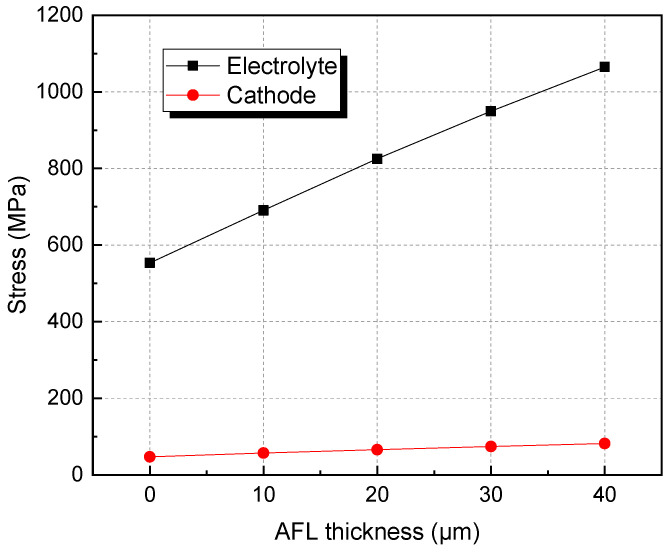
Stresses in electrolyte and cathode of SOFC with varying AFL thickness after re-oxidation.

Figure 5 shows the stresses in the electrolyte and cathode after re-oxidation with varying AFL thickness. The stresses in the electrolyte and cathode of the SOFC without an AFL are minimal. After the AFL thickness grows to 40 μm, the stresses in the electrolyte and cathode are almost one time larger than those without AFL because the expansion of the AFL becomes increasingly influential. Especially for the electrolyte, the stress is relatively large because of its thin thickness and large elastic modulus. According to Ref. [33], the cell has a maximum output power density with an AFL thickness of 9.8 μm. Therefore, an SOFC with an AFL thickness of about 10 μm is superior, considering its electrical performance and redox stability.

**Figure 6:**
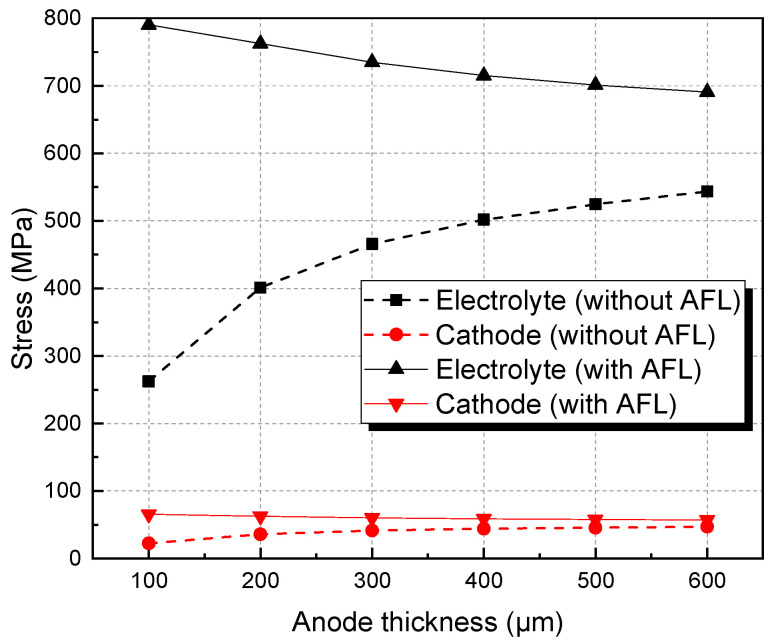
Stresses of electrolyte and cathode of SOFC with and without AFL as anode thickness changes.

Figure 6 shows the stresses in the electrolyte and cathode of SOFCs with and without an AFL after re-oxidation with varying anode thickness. For the SOFC without an AFL, the stresses of the electrolyte layer and the cathode layer are decreased with the reduction in anode thickness. In particular, when the thickness of the anode decreases to 100 μm, the stresses of the electrolyte and cathode are 262.46 and 22.47 MPa, respectively, and are less than their fracture strengths. However, for the SOFC with an AFL, by reducing the thickness of the anode substrate, the stresses of the electrolyte layer and cathode layer are increased but are still large enough to induce mechanical failure. Therefore, by adding an AFL, the effect of anode substrate thickness on the stresses of the electrolyte layer and cathode layer after re-oxidation is decreased, compared with an SOFC without an AFL. And the effect of anode thickness on the stress in the electrolyte and cathode gradually decreases after it reaches 400 μm.

## 4. Discussion

### 4.1. Model Validation

In order to validate the accuracy of the analytical model, the as-sintered residual stress of a half-SOFC model employed in Ref. [34] was calculated and compared with the results in Ref. [34]. The as-sintered residual stress in the electrolyte at room temperature (293 K) calculated by the analytical model was about 770 MPa, which is in accordance with the result (775 MPa) in Ref. [34], indicating that the analytical model is trustworthy in this work.

### 4.2. Results Discussion

**Figure 7:**
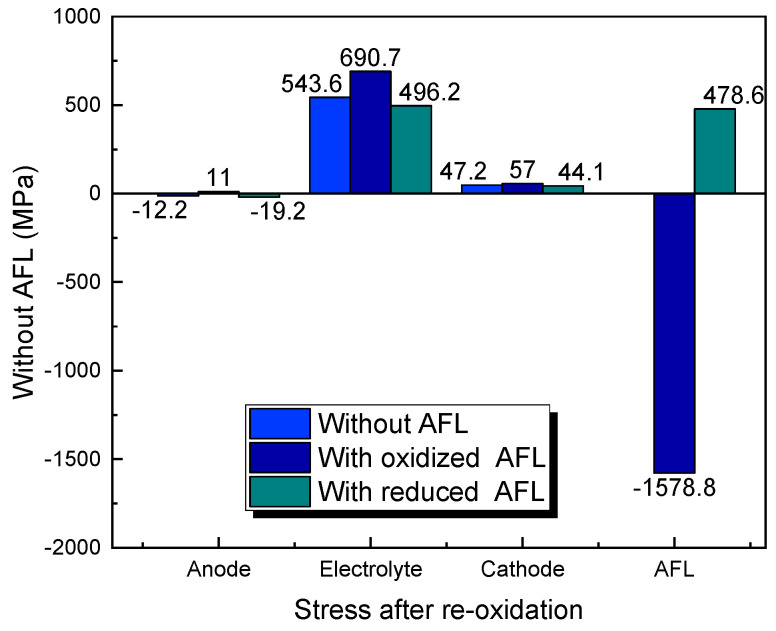
Stresses in SOFC after re-oxidation of anode substrate.

It is worth noting that the above-calculated results in this study are based on the assumption that the anode containing the substrate and AFL is completely oxidized. In fact, the anode oxidation may be inhomogeneous, which is gradually deepened along the thickness direction during the oxidation process [26]. When the anode substrate is fully oxidized, but the AFL is in a reduced state, the AFL contributes to the reduction in stresses in the electrolyte and cathode compared with the SOFCs without an AFL and with an oxidized AFL, as shown in Figure 7. The stress in the AFL changes from significant compressive stress to large tensile stress, compared with the SOFC with an oxidized AFL. The compressive stress in the anode substrate increases compared with the SOFC without an AFL but does not reach dangerous stress. In conclusion, although the AFL seriously increases SOFC failure stresses when fully oxidized, the AFL is still able to absorb part of the tensile stress caused by the oxidation of the anode substrate, thereby reducing the dangerous stresses of the electrolyte and cathode when the anode is partially oxidized.

**Figure 8:**
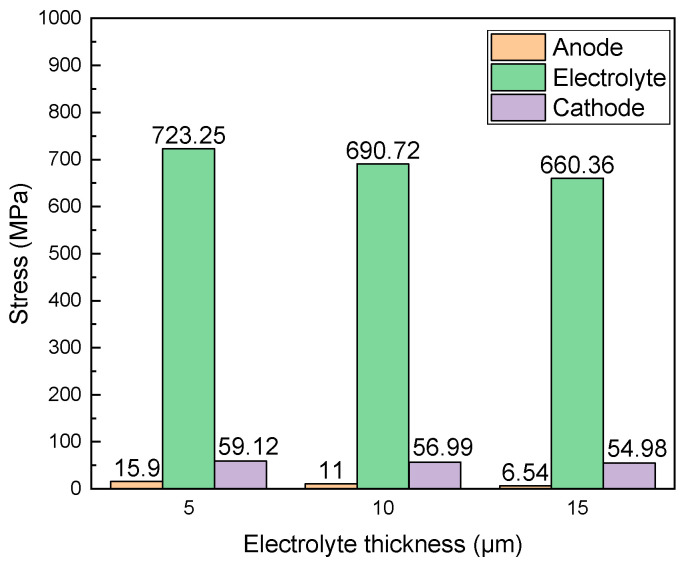
Stresses in the cell as the electrolyte thickness changes.

SOFCs are suitable for distributed power stations and are also an alternative for mobile power. For mobile applications, SOFCs are tolerant to carbon oxides and do not require extensive CO removal/gas-cleaning systems, as needed in PEMFCs [35]. The main challenges in SOFC applications are related to their operation at high temperatures, which requires long start-up times and is susceptible to severe thermal gradients and re-oxidation stresses. To mitigate these issues and improve stability, a variety of solutions have been proposed, such as developing new materials or metal-supported SOFCs [36]. Reducing the operating temperature to lower than 600 °C or to intermediate temperatures of 600–800 °C is one of the methods that can make SOFCs more practical devices. Mechanical degradation due to damage starts at about 0.5% cumulative redox strain, whereas macroscopic loss of integrity results when redox strain exceeds about 2.5% after seven cycles at 750 °C [37]. As the kinetics are strongly dependent on temperature, fast cooling of the stack with a rate of 3 °C min^−1^ or higher (between 600 and 800 °C) will slow the kinetics down sufficiently so that standard Ni-YSZ anode support cells can withstand the oxidation [38]. Reducing the thickness of the electrolyte membrane is one of the effective ways to lower the operating temperature and maintain the output performance [39]. Figure 8 shows the stresses in the cell after re-oxidation with varying electrolyte thickness. It can be seen that the stresses after re-oxidation in the cell increase, with the electrolyte thickness decreasing from 15 to 5 μm. This is because the dense electrolyte has higher strength, thus improving the overall resistance of the cell to damage. This research study provides a theoretical basis for the design of a reliable SOFC in the redox condition.

## 5. Conclusions

In this study, the influence of the anode structure on redox stability is discussed. And the effects of AFL and anode substrate thickness on the stress after re-oxidation are discussed to decrease the stresses in the electrolyte and cathode. This method provides a theoretical basis for designing reliable SOFCs under redox conditions in industrial applications. Based on the obtained results, the following conclusions can be made:

1. AFL re-oxidation has a great effect on the stress of the structure. By introducing an AFL on the anode substrate, the tensile stresses of the electrolyte and cathode of the SOFC with an AFL are increased by 27.07% and 20.77%, respectively, compared with the stresses of the SOFC without an AFL after the full re-oxidation of the anode.
2. After re-oxidation, the stresses of the electrolyte layer and cathode layers are increased with increasing AFL thickness. The stresses in the electrolyte and cathode with a 40 μm AFL are almost one time larger than those without an AFL. An SOFC with an AFL thickness of about 10 μm is superior for better electrical performance and redox stability.
3. The thickness of the anode substrate plays a more important role in the SOFC without an AFL than in the SOFC with an AFL. By increasing the thickness of the anode substrate, the stresses in the electrolyte and cathode decrease.

## Figures and Tables

**Table 1:** Dimensions and materials for the typical anode-supported SOFC [10].

Component	Anode	Electrolyte	Cathode
Thickness (m × 10^−6^)	600	10	40
Material	NiO/Ni-YSZ	YSZ	LSCF

**Table 2:** Material properties [23,24,25,26].

	Temperature (°C)	Ni-YSZ	NiO-YSZ	AFL	YSZ	LSCF
Young’s modulus (GPa)	25		106	153	200	10
	800	64.769	101	129 110.8845	157	10
Thermal expansion coefficient (°C^−1^ × 10^−6^)	25		11.7	9.65	7.6	8.8
	800	12.41	12.41	11.205	10	12.84
Poisson’s ratio	25		0.301	0.3	0.31	0.3
	800	0.287	0.3	0.3	0.31	0.3

**Table 3:** Fracture strengths of SOFC materials after re-oxidation of anode [31].

Material	Temperature (°C)	NiO-YSZ	YSZ	LSCF
Fracture strength (MPa)	25	187	232	52
	800	124	154	75

## Data Availability

Data are contained within the article.

## Nomenclature

**Abbreviation**	
SOFCs	solid oxide fuel cells
AFL	anode functional layer
CTE	thermal expansion coefficient
**Symbols**	
*E_i_*	Young’s modulus, MPa
*υ_i_*	Poisson’s ratio, -
*α_i_*	thermal expansion coefficient, °C^−1^
T	temperature, °C
σ*_i_*	stress, MPa
*ε_i_*	strain, -
*k*	curvature, -
*t_i_*	thickness, m
**Subscripts and superscripts**	
*s*	anode substrate
*f*	AFL
*e*	electrolyte
*c*	cathode
*ox*	oxidation
*th*	thermal expansion
